# Restorative resection of radiation rectovaginal fistula can better relieve anorectal symptoms than colostomy only

**DOI:** 10.1186/s12957-017-1100-0

**Published:** 2017-02-02

**Authors:** Qinghua Zhong, Zixu Yuan, Tenghui Ma, Huaiming Wang, Qiyuan Qin, Lili Chu, Jianping Wang, Lei Wang

**Affiliations:** 1 Department of Colorectal Surgery, The Sixth Affiliated Hospital of Sun Yat-sen University, Guangzhou, Guangdong Province China; 2 Clinical Research Division, Fred Hutchinson Cancer Research Center, University of Washington, Seattle, WA USA; 3 Guangdong Provincial Key Laboratory of Colorectal and Pelvic Floor, Sun Yat-sen University, No.26, Road Yuancun ErHeng Road, Tianhe District, Guangzhou, 510655 China

**Keywords:** Radiation-induced intestinal injury, Rectovaginal fistula, Restorative resection, Colostomy

## Abstract

**Background:**

Radiation-induced rectovaginal fistula (RVF) is a severe and difficult complication after pelvic malignancy radiation. This study was to retrospectively compare the outcomes of restorative resection and colostomy only in remission of anorectal symptoms.

**Methods:**

We enrolled a cohort of 26 consecutive cases who developed RVF after pelvic radiation. Two main procedures for these patients in our institution were used: one was restorative resection and pull-through coloanal anastomosis with a prophylactic colostomy, and another was a simple colostomy without resection. Thus, we divided these patients into these two groups. Anorectal symptoms including rectal pain, bleeding, tenesmus, and perineal mucous discharge were recorded and scored prior to surgery and at postoperative multiple time points.

**Results:**

The baseline was similar among the two groups. All patients acquired good efficacy with improved symptoms at postoperative 6, 12, and 24 months, when compared to baseline. In addition, the resection group showed a better remission of tenesmus (6 months 33.3 vs 0%; 12 months 66.7 vs 16.7%) and perineal mucous discharge (6 months 88.9 vs 6.7%; 12 months 77.8 vs 15.4%; 24 months 85.7 vs 25.0%). Furthermore, three (30%) patients in the resection group successfully reversed stomas while no stoma was closed in the simple colostomy group.

**Conclusions:**

Both restorative resection procedure and colostomy only can improve anorectal symptoms of radiation-induced RVF, but restorative resection can completely relieve anorectal symptoms in selected cases.

## Background

Rectovaginal fistula (RVF) is one of the most severe complications after radiation therapy for pelvic malignancy. Patients with RVF usually present with passage of air, stool, or even purulent discharge from the vagina [1–4]. In addition, patients also suffer from refractory anorectal symptoms of radiation proctitis, such as rectal bleeding, perianal pain, and tenesmus [5, 6]. Quality of life was thus severely impaired in these patients [7]. Notably, life expectancy is good for these RVF patients if no tumor relapsed. Therefore, how to control anorectal symptoms and improve quality of life is with great clinical value in both research and practice.

However, radiation-induced RVF remains a challenge [8]. Radiotherapy can not only damage normal anatomy and biological function but also lead to poor healing in the following reconstructive process [9]. A diverting stoma for these RVFs is preferred by many surgeons [10, 11]. The reasons include challenges of surgical techniques, high failure rates (75%) [8], and high mortality rates (30%) for other aggressive procedures [12, 13]. Whether leaving the damaged bowel in place would relieve symptoms or not is still unclear. The procedure that resected the mucosa of rectal lesion segment and pull down the normal bowel to make a coloanal anastomosis (Park’s procedure) is a classic option for extreme low rectal cancer after neoadjuvant chemoradiotherapy [14–17]. Based on our extensive experience in Park’s procedure for rectal cancer, we tried a similar procedure but without mucosa resection for some radiation-induced RVFs in our center (the resection group). Meanwhile, other patients still received a simple colostomy to divert the stool (the diversion group). This study was to retrospectively compare the outcomes of two specific surgical options in controlling local anorectal symptoms of radiation-induced RVFs.

## Methods

### Patient enrollment

**Fig. 1:**
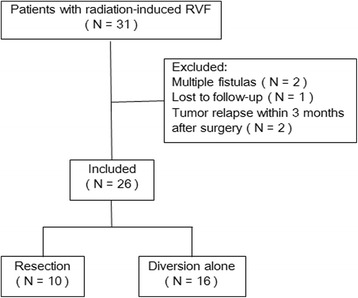
Patient enrollment flow

A total of 31 patients who underwent restoration resection of damaged bowel or simple colostomy for radiation-induced RVF were retrospectively included at the Sixth Affiliated Hospital, Sun Yat-sen University (SYSU) of China between Jan 2012 and Aug. 2015. Exclusion criteria: (1) multiple fistulas including vesico-enteric fistula (*n* = 2), (2) lost to follow-up (*n* = 1), and (3) tumor relapse within 3 months after surgery (*n* = 2) (Fig. 1). Finally, after exclusion, a cohort of 26 patients were enrolled in this study. Resection of damaged bowel with prophylactic colostomy was performed in ten cases and a simple colostomy was performed in the remaining 16 cases. The study was approved by the ethical committee of the Sixth Affiliated Hospital of SYSU and met the guidelines of local responsible governmental agency. Due to the retrospective nature of the study, informed consent was waived.

### Symptoms scores, surgical indications, and follow-up

Symptoms of radiation-induced RVF were graded by the modified radiation proctopathy system assessments scale (RPSAS) (Table 1) [18]. According to this scale, 1 point represents free of a specific symptom. A score that was 1 point (no problem) after operation was considered a complete remission. In addition, the Wexner continence grading scale was used after the stoma was reversed [19].

**Table 1:** Symptom scores by modified radiation proctopathy system assessments scale (RPSAS)

Severity
1. No problem
2. Mild problem—can be ignored when you do not think about it
3. Moderate problem—cannot be ignored; no effect on daily activities
4. Severe problem—influences your concentration on daily activities
5. Very severe problem—markedly influences your daily activities and/or requires rest
Frequency
1. Monthly
2. Weekly
3. Several times per week
4. Daily
5. Throughout the day
Symptoms
Rectal pain
Rectal bleeding
Tenesmus
Perineal mucous discharge^a^

The RPSAS score for the patient without a specific symptom was 1 point

^a^Perineal mucous discharge included discharge from vagina and/or rectum

Radiation-induced RVF remains one challenge, the choice of procedure had to be decided by doctors and patients together. The patients should be well informed of the advantages and disadvantages of the two different procedures without bias before the surgery. (1) Technically, a colostomy is a relatively simple and safe procedure. However, spontaneous healing of fistula is rare and it would be hard for patients to reverse the stoma. In addition, whether leaving the damaged bowel in place would relieve symptoms or not is still unclear. (2) Patients enrolled in the resection group should be in good general condition (ASA grade I or II) and had no evidence of metastatic disease. Patients should be clearly informed that the operation may lead to many postoperative complications and it is difficult to restore bowel continuity in a short term.

Follow-up was scheduled at 3, 6, 12, 24, and 36 months after operation. If tumor relapsed, no further follow-up was conducted.

### Procedure protocols

In the resection group, damaged rectal segment was resected, then a pull-through coloanal anastomosis was performed, and a colostomy was created, while the simple colostomy group underwent only a colostomy. The patient was in general anesthesia and placed in the Trendelenburg position. This procedure can be conducted by laparoscopy or laparotomy, which depends on the surgeon’s experience. In the abdominal phase, the mobilization of the left colon began by identification and high ligation of the inferior mesenteric artery and vein at the sacral promontory. The left ureter was clearly visible and protected before the ligation. The inferior mesenteric pedicle was ligated and dissected. We preferred a medial to lateral manner to mobilize the left colon. The splenic flexure was also completely mobilized, to ensure the length to pull down proximal normal colon for the anastomosis without tension. The rectum was then dissected and mobilized in both lateral and posterior surfaces until the levator ani level. The dissection is very close to the rectal wall to minimize adjacent tissue resection. During this procedure, a rigid and narrowed pelvis would be observed because of extensive pelvic fibrosis after radiation.

In the perineal phase, the anus was fully exposed by a self-produced circle anal retracter. A purse-string suture was inserted 1–2 cm above the dentate line (below the inferior border of the fistula). Then the operator made a circumferential full-thickness incision at the level of just below the suture line. Perirectal dissection should be minimized in order to preserve neighboring vital structures such as sphincters. The surgeon performed the dissection with one hand; meanwhile, the surgeon inserted fingers of the other hand into the rectum and vagina, which guided the process of detaching the rectum and anal canal from the vagina. After full mobilization, the rectum was pulled out through the anus. The rectum was traversed by a linear stapler or hand. Thus, the lesion rectal segment was removed and the specimen was sent to one pathologist to evaluate. The proximal healthy appearing, non-irradiation damaged, and rich-vascularized sigmoid colon was sutured to the anus by a stapler or manually to perform a reliable coloanal anastomosis. Colon tension or torsion should be avoided before the anastomosis. A transverse loop colostomy, which details were described in our previous study [20], was then created and a presacral drainage was placed. Stoma reversal was scheduled at least 6 months after the procedure, if no contraindication in rectal examination was found. In the colostomy only group, only transverse loop colostomy was performed.

### Statistical analysis

Continuous variables were compared by using the Student *t* test. The chi-square test or Fisher’s exact test was used to compare categorical variables. Non-parameters such as symptom scores were analyzed by Wilcoxon signed-rank test. Kruskal-Wallis was used to compare preoperative ASA grade. A two-sided *P* < 0.05 was considered to be statistically significant. All statistical analyses were performed by SPSS version 20 (SPSS, Inc., Chicago, USA).

## Results

### Baseline characteristics

Demographic and clinical data are shown in Table 2. All 26 patients were treated with radiation therapy for gynecological malignancies, among which 76.9% were cervical cancer. These patients received external-beam radiotherapy (40–60 Gy in 20–30 fractions) and intracavitary radiotherapy (24–54 Gy in 4–9 fractions). The baseline characteristics of enrolled patients in both groups are almost comparable. There were no differences in primary cancer, age, time from the end of radiotherapy to fistula formation, fistula location, and fistula size between the groups (Table 2). In addition, both groups had comparable symptoms before the surgery, including rectal pain, bleeding, tenesmus, and perineal mucous discharge. However, preoperative ASA score is different (*P* < 0.05), which revealed better general condition before surgery in resection group, comparing to the simple colostomy group.

**Table 2:** Baseline characteristics in two groups

	Resection group	Simple colostomy	*P*
	(*n* = 10)	(*n* = 16)	
Primary cancer			0.256
Cervix	8 (80.0%)	12 (75.0%)	
Endometrium	1 (10.0%)	4 (25.0%)	
Vagina	1 (10.0%)	0	
Age at surgery (years)	55.0 (44–72)	57.5 (39–67)	0.590
Time from the end of RT to fistula formation (months)	14.5 (11–25)	12 (6–120)	0.600
Preoperative ASA grade			0.015
ASA I	2 (20.0%)	0	
ASA II	8 (80.0%)	11 (68.8%)	
ASA III	0	5 (31.2%)	
Fistulas			
Location (distance from anus, cm)	4.0 (2.5–5.0)	4.0 (2.0–9.0)	0.598
Size (diameter, cm)	1.2 (0.2–4.0)	1.5 (0.3–3.0)	0.754
Rectal pain scores	8 (1–10)	9 (1–10)	0.551
Rectal bleeding scores	6 (1–9)	6.5 (1–9)	0.201
Tenesmus scores	9.5 (8–10)	8.5 (1–10)	0.087
Perineal mucous discharge scores	8 (6–10)	8 (6–10)	0.121

### Symptoms improvement

**Fig. 2:**
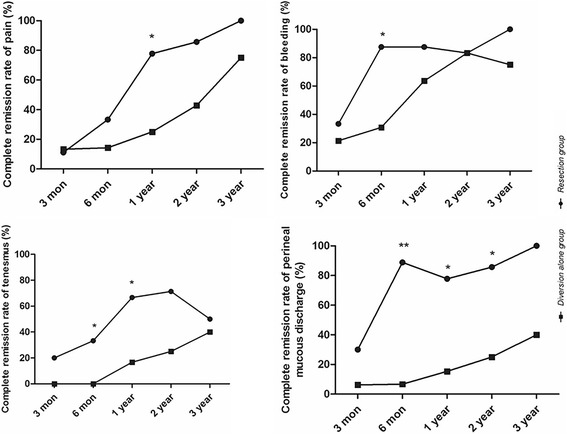
Complete remission rate of individual symptom for two groups (*P < 0.05, **P < 0.01)

In total, before the surgery, the proportions of patients who experienced severe pain, severe bleeding, severe tenesmus, and perineal discharge were 73.1% (19/26), 19.2% (5/26), 76.9% (20/26), and 88.5% (23/26), respectively. Individual symptom scores including rectal pain, rectal bleeding, tenesmus, and perineal mucous discharge decreased significantly at 3 months, and the efficacy persisted until postoperative 2 years in both groups, when compared with the baseline. In addition, when compared with the simple colostomy group, the resection group showed a better complete remission rate of tenesmus (6 months 33.3 vs 0%, *P* < 0.05; 12 months 66.7 vs 16.7%, *P* < 0.05) and perineal mucous discharge (6 months 88.9 vs 6.7%, *P* < 0.001; 12 months 77.8 vs 15.4%, *P* < 0.01; 24 months 85.7 vs 25.0%, *P* < 0.05) (Fig. 2).

### Colostomy reversal

In resection group, no death was observed during the follow-up. Three patients (30.0%) had successful closure of the temporary colostomy (two reversals at postoperative 9 months and the other one at postoperative 35 months). All of these three patients recovered good intestinal continence after ostomy reversal with a Wexner score of 4, 0, and 0 points, respectively. A small and localized asymptomatic anastomotic leak was detected on routine colonoscopy in three (30%) patients, who were under surveillance without intervention. Interestingly, the anastomotic leak healed in one of these three points and then successfully underwent stoma reversal at postoperative 35 months. The remaining six patients (60.0%) had delayed stoma reversals or preferred permanent stoma for various reasons: anastomotic leak (2 points), mild incontinence by anorectal manometry and defecography (2 points), and two remaining patients preferred permanent stoma because of satisfaction with current condition.

In simple colostomy cohort, three patients (18.8%) had primary tumor recurrence during follow-up (at postoperative 11, 15, and 18 months). No spontaneous healing of RVF was observed through coloscopy during follow-up, and none of these 16 patients reversed the stomas in this group.

## Discussion

Radiation-induced RVF often presents anorectal symptoms including perineal discharge, bleeding, pain, and tenesmus. RVF can also lead to psychiatric problems and impair social activities due to these intractable symptoms in women. Our results also confirm the huge negative effect on quality of life, when evaluated by objective complaint and subjective scoring scale. Radiotherapy is like a sword with two blades. On one side, radiotherapy can cure the tumor and benefit patients with a longer life expectancy. On the other side, radiation-induced RVF will develop and potentially continue to increase, as more radical radiotherapy regimens are used, and thus greatly impact cancer survivor’s quality of life in the prolonged lifetime [21].

A diverting stoma is preferred by some surgeons for its simplicity and relative low morbidity for radiation-induced RVF [2, 16, 22]. However, according to our previous experience and this study, ostomy does not obtain satisfaction in controlling tenesmus and perineal discharge. Consistent with previous studies, we also confirm that diverting the stool stream can improve some symptoms, such as pain, bleeding, tenesmus, and drainage, but cannot eliminate these symptoms, because the rectal lesion still exists and this fundamental issue is not resolved [23, 24]. Furthermore, Piekarski et al. described a cohort of 17 cases with patients who only underwent fecal diversion. In three patients, spontaneous healing of rectovaginal fistula occurred, but no patient had her abdominal stoma closed [25]. In our cohort of 16 cases who underwent simple colostomy, no spontaneous healing of fistula was observed. Similarly, no patient in this group reversed the ostomy.

To minimize the injury of extensively dissecting poor healing tissues in the rigid and fibrotic narrowed pelvis, lesion rectal segment resection and pull-through coloanal anastomosis is regarded to be one advanced invasive procedure for radiation-induced RVF [13, 26]. However, considerable morbidity of this procedure is documented, including high risks of anastomotic leaks [27]. Our hospital is the largest tertiary center with referral of radiation proctitis mainly from south China. Apart from ostomy alone, we perform this complicated procedure under strict indication by the same experienced surgeon. Patients should be in good general condition (ASA grade I or II) and no evidence of metastatic disease found. A temporary prophylactic colostomy is essential. The key points of the surgical procedure are as follows: (a) complete mobilization of the splenic flexure to obtain adequate length of colon for restoration; (b) close dissection to the bowel downward until the pelvic floor was reached, in order to minimize adjacent tissue damage; (c) subtotal rectal resection above the level of the fistula; (d) pull-through anastomosis could be done by hand-sewn or stapled methods, and the proximal segment should be apparently healthy, nonirradiated, and well-vascularized; and (e) maintenance of an adequate axis and colon tension for reconstruction.

Parks et al. firstly reported mucosa dissection of the rectum followed by pull-through coloanal anastomosis for radiation-induced RVF in five patients. Among them, one patient with intractable perianal pain acquired complete relief of pain after this procedure [26]. Similarly, Gazet et al. showed this procedure can help patients get rid of anorectal symptoms of radiation proctitis including perianal pain [17]. In our study, we provide more evidence that this procedure can eliminate tenesmus and perineal discharge. Furthermore, resection of damaged bowel can improve quality of life greatly, even in the condition of an unclosed stoma.

Do et al. found that microvascular damage caused by radiation can not only cause the symptoms of radiation proctitis but also impair healing capacity of these irradiated tissues [24]. Thus, anastomotic leaks can occur even after a perfect anastomosis [27]. Most studies reported poor outcomes with a high incontinence rate from 10 to 50% after stoma reversal. Thus, many patients return to a permanent colostomy after incontinence occurs [13, 16, 28]. Cooke et al. reported variable severity of incontinence, which presents persisted liquid-like stool in seven of nine patients who underwent low anastomosis at dentate line level in these patients with radiation fistulas [27]. In our study, two patients presented with variable changes in anal resting tone, squeeze pressure, decreased rectal volume, or rectal compliance by anorectal manometry and defecography at postoperative 12 months, which indicates high risk of incontinence after stoma reversal. After informing the patients these risks, both patients decided to delay stoma closure. Our study was also limited by a nonrandomized, retrospective design and a relative small sample size. Therefore, large cohort and prospective studies are needed to confirm our findings.

## Conclusions

In conclusion, both restorative resection procedure and colostomy only can improve anorectal symptoms of radiation-induced RVF. But resection can better relieve anorectal symptoms and offer an opportunity of restoration of bowel continuity without fatal complications. For patients who are suffering from severe symptoms of radiation proctitis, or want to restore bowel continuity, resection of the damaged bowel could be recommended.

## Abbreviations

RPSAS: Radiation proctopathy system assessments scale
RVF: Rectovaginal fistula
